# Experimental and Mechanistic Study on Flotation Separation of Chalcopyrite and Molybdenite Using the Novel Depressant 2-Mercapto-6-Methylpyrimidin-4-ol

**DOI:** 10.3390/molecules30061396

**Published:** 2025-03-20

**Authors:** Xiangwen Lv, Anruo Luo, Xiong Tong, Jianhua Chen, Sheng Jian

**Affiliations:** 1 Faculty of Land and Resources Engineering, Kunming University of Science and Technology, Kunming 650093, China; lv_xiangwen@163.com; 2 Kunming Metallurgical Research Institute Co., Ltd., Kunming 650031, China; jiansheng545@163.com; 3 School of Chemistry and Chemical Engineering, Guangxi University, Nanning 530004, China; 4 School of Resources, Environment and Materials, Guangxi University, Nanning 530004, China; jhchen@gxu.edu.cn; 5 Guangxi Key Laboratory of Processing for Non-Ferrous Metal and Featured Materials, Guangxi University, Nanning 530004, China

**Keywords:** chalcopyrite, molybdenite, flotation, depressant, DFT

## Abstract

Chalcopyrite and molybdenite are vital strategic metal resources. Due to their close association in ores, flotation methods are commonly used for separation. The flotation separation method primarily employs the “copper depression and molybdenum flotation” process, enhancing the wettability difference between chalcopyrite and molybdenite through a chalcopyrite depressant. Traditional depressants often face challenges, including low selectivity, high dosage requirements, poor stability, and significant environmental pollution, highlighting the need for new, highly selective green reagents. This study introduces the novel chalcopyrite depressant 2-mercapto-6-methylpyrimidin-4-ol (MMO) for flotation separation. The influence of MMO on chalcopyrite and molybdenite flotation recovery was examined through microflotation experiments. Additionally, the effects of MMO and ethyl xanthate on surface wettability were assessed via contact angle measurements. The adsorption microstructure and interaction mechanism of MMO on chalcopyrite were elucidated using FT-IR, TOF-SIMS, and XPS analyses and DFT simulations. Results indicate that MMO enhances chalcopyrite hydrophilicity and exhibits a strong depressing effect on its flotation, while minimally impacting molybdenite recovery. Thus, it serves as an effective depressant. During adsorption, N and S atoms in MMO donate electrons to Fe and Cu ions, leading to triple bond adsorption and a stable chelate structure. These findings are crucial for achieving a greener and more efficient flotation separation of copper and molybdenum.

## 1. Introduction

Molybdenum is a crucial strategic metal resource known for its excellent properties, including a low expansion coefficient, high electrical conductivity, and good thermal conductivity. It is extensively utilized across various industries, such as metallurgy, electrical engineering, chemical manufacturing, and aerospace. Molybdenite serves as the primary source of molybdenum metal [1,2]. As resource development and utilization continue, high-quality molybdenum resources are becoming increasingly depleted. Consequently, poor-quality, fine, and mixed porphyry copper–molybdenum ores have emerged as significant sources of molybdenum [3,4,5]. Reports indicate that nearly half of the world’s molybdenum reserves are contained within porphyry copper deposits [6]. Given that molybdenum and copper are often closely associated in ores [7] and share similar specific gravities while being non-magnetic, flotation is commonly employed for their separation in practice [8]. Flotation is a mineral processing method that exploits differences in the wettability of mineral surfaces to achieve separation. During the flotation process, collectors enhance the hydrophobicity of certain minerals, while depressants increase the hydrophilicity of others, allowing hydrophobic minerals to concentrate at the gas–liquid interface while hydrophilic minerals remain in the aqueous phase [9].

Currently, the separation of copper and molybdenum primarily employs the “copper depression and molybdenum flotation” process, which effectively separates these metals through the addition of copper mineral flotation depressants [10,11]. Traditional copper mineral depressants can be categorized into two types: inorganic and organic depressants. Among inorganic depressants, cyanides such as potassium cyanide, sodium cyanide, and ferrocyanide are widely used globally [12]. These reagents are effective at low dosages and demonstrate excellent depressing performance during the concentration stage with high selectivity [13,14]. However, cyanide is a highly toxic substance that poses significant risks to human health and severely contaminates the environment. Due to increasing environmental protection pressures, its use has been strictly regulated, prompting many mineral processing plants to seek alternative depressants.

Sulfides, such as sodium sulfide and sodium hydrosulfide [15,16], are also commonly used depressants in the copper–molybdenum separation process, with sodium sulfide being the most frequently employed reagent [17]. It is cost-effective and demonstrates good reagent removal effects, making it widely utilized in industrial copper–molybdenum separation. However, sodium sulfide releases sulfur-containing ions during flotation and can decompose under acidic conditions, producing hydrogen sulfide gas, which poses significant environmental risks [18].

Other types of inorganic depressants, known as Knox reagents, are less commonly used [19]. This category includes phosphorus Knox and arsenic Knox reagents. Their mechanism involves the adsorption of thiophosphate or thioarsenate onto the surfaces of copper minerals, forming hydrophilic copper thiophosphate or copper thioarsenate, which depresses collector adsorption and prevents the flotation of copper minerals. Knox reagents have a slower oxidation rate compared to sodium sulfide and exhibit stronger depressing effects, allowing for lower dosages. However, the preparation of these reagents involves arsenic, which is highly toxic, and the production process generates H_2_S gas, leading to environmental pollution and potential explosion hazards. Consequently, their use has not gained widespread acceptance.

Thiol-based small molecules are the most common organic depressants for copper minerals. Among these, thioglycolic acid-based compounds exhibit strong depressing properties for copper minerals. Their thiol and carboxyl groups can form robust bonds with the copper atoms in these minerals, rendering the copper mineral surfaces hydrophilic and thereby depressing flotation [20]. However, in practical applications, the effectiveness of thiol-based depressants can be compromised by other ore components, and the stability of some thiol-based depressants requires enhancement.

Thiourea-based organic depressants interact with metal ions on the surfaces of copper minerals through the thiourea group in their molecular structure, forming stable complexes that increase the hydrophilicity of the copper mineral surfaces and achieve a depressing effect [21,22]. Nevertheless, the selectivity of thiourea-based depressants is sometimes suboptimal; while they depress copper minerals, they can also adversely affect molybdenite, and their stability and efficiency may vary under different slurry conditions.

Sulfonic acid-based organic depressants can also adsorb onto the surfaces of copper minerals, depressing their flotation while demonstrating some selectivity and depressing capacity [23,24]. However, in practical applications, these sulfonic acid depressants often exhibit poor adaptability to various ores, leading to less than ideal depressing effects on complex ores. Additionally, there are challenges associated with cost control when utilizing these depressants on a large industrial scale.

Currently, traditional depressants often face issues such as low selectivity, high dosage requirements, poor stability, elevated costs, and significant environmental pollution. To address the aforementioned challenges, this study proposes a novel inhibitor development strategy based on molecular functional design. For the first time, the small-molecule organic reagent 2-mercapto-6-methylpyrimidin-4-ol (MMO) was applied as a chalcopyrite flotation depressant. The innovation of MMO lies in three key aspects: (1) the synergistic interaction between its thiol group and pyrimidine ring enhances specific binding to copper mineral surfaces [25]; (2) the rigid structure of the pyrimidine ring improves adsorption stability; and (3) hydrogen bonding between the hydroxyl group and water molecules increases hydrophilicity post-adsorption. Microflotation experiments systematically investigated the effects of MMO dosage and pH on copper and molybdenum recovery rates. Combined with contact angle measurements to elucidate surface wettability modulation mechanisms, multiscale characterization techniques including FT-IR, TOF-SIMS, and XPS were employed. Density functional theory (DFT) calculations further revealed the adsorption configuration [26,27], electron transfer pathways, and energy evolution at the chalcopyrite–MMO interface, establishing a quantitative structure–activity relationship of “molecular structure–interface behavior–separation performance”. Results demonstrate that MMO achieves efficient chalcopyrite depression at low concentrations (6 × 10^−4^ mol/L), reducing its recovery rate to approximately 10%, while minimally affecting molybdenite (recovery > 70%). This superior performance originates from (1) strong covalent bonding between sulfur/nitrogen atoms in MMO and copper atoms, forming a stable tridentate chelation structure, and (2) enhanced hydrophilicity through hydroxyl-mediated hydrogen bonding. This research provides a paradigm for green reagent design and mechanistic exploration in copper–molybdenum separation, offering significant potential for improving strategic metal resource utilization.

## 2. Results and Discussion

### 2.1. Microflotation Experiments

**Figure 1:**
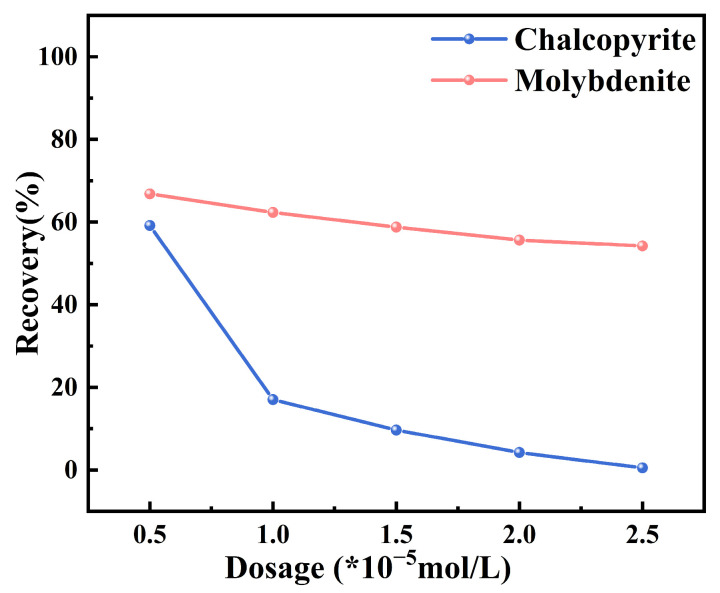
The effects on the flotation behaviors of chalcopyrite and molybdenite under various depressant dosages without collector.

Figure 1 illustrates the effect of different depressant dosages on the flotation recovery of chalcopyrite and molybdenite under collector-free conditions, with experiments conducted at room temperature and a fixed pH of approximately 7. At an MMO dosage of 0.5 × 10^−5^ mol/L, the chalcopyrite recovery rate is 59.12%. As the MMO dosage increases, the recovery rate of chalcopyrite continues to decline; it drops to 17.03% at 1 × 10^−5^ mol/L, and then the rate of decline begins to decrease. When the MMO dosage reaches 2.5 × 10^−5^ mol/L, the chalcopyrite recovery rate falls to 0.50%. In contrast, the flotation recovery rate of molybdenite is less affected by the MMO dosage. At 0.5 × 10^−5^ mol/L, the recovery rate for molybdenite is 66.82%, and it decreases to 54.21% when the dosage is increased to 2.5 × 10^−5^ mol/L. From 0.5 × 10^−5^ mol/L to 2.5 × 10^−5^ mol/L, the flotation recovery rate of chalcopyrite decreases by 58.62%, while the recovery rate of molybdenite only decreases by 12.61%. This indicates that MMO can selectively depress chalcopyrite, making it a viable flotation depressant for copper–molybdenum separation. The experimental results were subjected to error analysis and significance tests, with the quantitative outcomes summarized in Table 1 and Table 2. All datasets exhibited relatively small standard deviations (s < 2.63), confirming the satisfactory repeatability of the experimental procedures. Furthermore, statistically significant differences in recovery rates between chalcopyrite and molybdenite were observed across all MMO dosage levels (*p* < 0.05), demonstrating the compound’s effective selectivity in flotation separation of these minerals under collector-free conditions.

**Figure 2:**
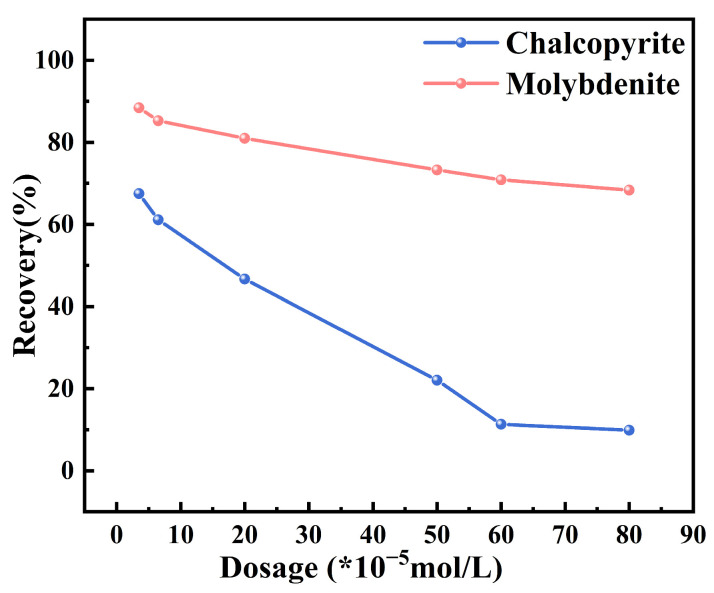
The effects on the flotation behaviors of chalcopyrite and molybdenite under various depressant dosages.

To better assess the actual application effect of MMO in chalcopyrite flotation, an MMO dosage experiment was conducted using 1 × 10^−5^ mol/L ethyl xanthate as a collector, based on the experimental conditions shown in Figure 1. The results, presented in Figure 2, indicate that under collector conditions, the chalcopyrite recovery rate continued to decline with increasing MMO dosage, although the depressing effect on chalcopyrite flotation diminished at lower dosages. When the dosage reached 2 × 10^−4^ mol/L, the chalcopyrite flotation recovery rate dropped to 46.68%. At 6 × 10^−4^ mol/L, the recovery rate fell further to 11.31%, and, at 8 × 10^−4^ mol/L, it slightly decreased to 10.13%, showing a significantly reduced rate of decline. The flotation recovery rate of molybdenite increased notably in the presence of the collector and was relatively less affected by MMO. Even at an MMO dosage of 8 × 10^−4^ mol/L, the molybdenite recovery rate remained at 70.84%. This demonstrates that while MMO has a significant impact on the effectiveness of the collector in chalcopyrite flotation, it exerts less influence on the collector’s effectiveness in molybdenite flotation. Statistical analysis of experimental errors and significance testing were conducted, with detailed results presented in Table 3 and Table 4. All datasets exhibited consistently low standard deviations (s < 2.4), confirming the high repeatability of the experimental procedures. Notably, statistically significant differences in recovery rates between chalcopyrite and molybdenite were observed across all tested MMO dosages (*p* < 0.001). These findings conclusively demonstrate that MMO retains effective selectivity in the flotation separation of chalcopyrite and molybdenite even when ethyl xanthate is employed as the collector.

**Figure 3:**
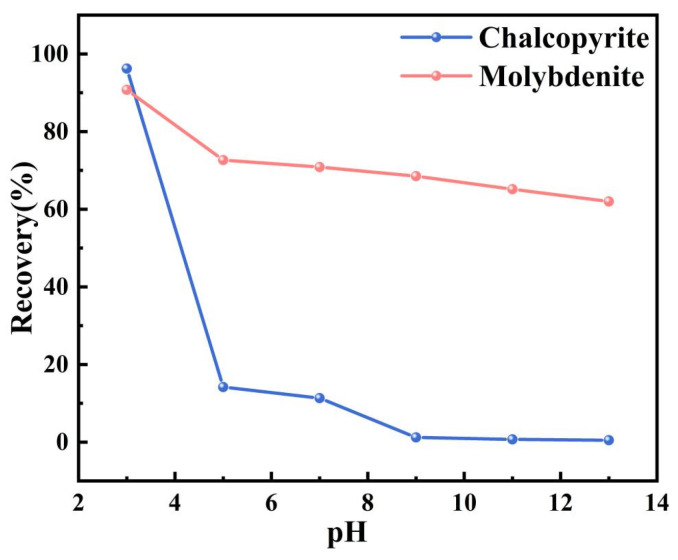
The effects on the flotation behaviors of chalcopyrite and molybdenite under various pH levels.

Figure 3 presents the recovery curve for chalcopyrite and molybdenite flotation using MMO as a depressant and ethyl xanthate as a collector at different pH values. The experiments were conducted at room temperature, with a fixed MMO dosage of 6 × 10^−4^ mol/L and an ethyl xanthate dosage of 1 × 10^−5^ mol/L. At a pH of 3, the flotation recovery rate of chalcopyrite reaches 96.21%, while molybdenite achieves a recovery rate of 90.75%. Under these conditions, MMO has almost no depressing effect on chalcopyrite. However, as the pH rises to 5, the flotation recovery rate of chalcopyrite sharply declines to 14.17%, and the recovery rate of molybdenite drops to 72.64%. At a pH of 7, the recovery rates are 11.31% for chalcopyrite and 70.84% for molybdenite. When the pH increases to 9, the recovery rate of chalcopyrite further decreases to 1.19%, while molybdenite’s recovery rate falls to 68.49%. As the pH continues to rise, the flotation recovery rates for both minerals decrease slightly. MMO effectively depresses chalcopyrite flotation across a wide pH range, demonstrating stronger depression in alkaline conditions and maintaining a relatively good depressing effect in less acidic environments (pH > 5). This indicates that MMO has significant application potential as a chalcopyrite depressant for copper–molybdenum flotation separation. Statistical error analysis and significance testing of the experimental results are presented in Table 5 and Table 6. The analysis revealed consistently low standard deviations across all datasets (s < 1.03), confirming the high reproducibility of the experimental procedures. Notably, under pH >3 conditions, statistically significant differences in recovery rates between chalcopyrite and molybdenite were observed (*p* < 0.001). This demonstrates that MMO exhibits pronounced selective separation efficacy for these minerals within this specific pH range.

### 2.2. Contact Angle Measurements

**Figure 4:**
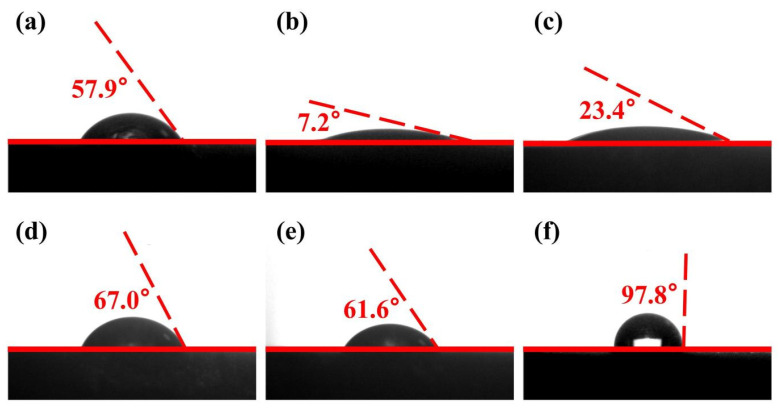
The results of contact angle measurements: (**a**) untreated chalcopyrite samples; chalcopyrite samples treated by (**b**) MMO and (**c**) ethyl xanthate and MMO; (**d**) untreated molybdenite samples; and molybdenite samples treated by (**e**) MMO and (**f**) ethyl xanthate and MMO.

The surface wettability of chalcopyrite and molybdenite samples—untreated, treated with the depressant MMO, and treated with both the collector ethyl xanthate and MMO—was characterized through contact angle measurement analysis, as shown in Figure 4. The contact angle for the untreated chalcopyrite sample was 57.9°, while the molybdenite sample had a contact angle of 67.0°. After treatment with the depressant MMO, the chalcopyrite sample’s contact angle decreased to 7.2°, whereas the molybdenite sample’s contact angle was 61.6°. When the chalcopyrite sample was treated with both the collector ethyl xanthate and MMO, the contact angle increased to 23.4%, while the molybdenite sample exhibited a contact angle of 97.8°. For chalcopyrite, MMO adsorbs onto its surface, enhancing hydrophilicity and diminishing the collector’s effectiveness. In contrast, MMO has minimal impact on the surface hydrophobicity of molybdenite and does not significantly affect the performance of ethyl xanthate. This sequence of treatments leads to a marked increase in the hydrophilicity of the chalcopyrite surface and a significant increase in the hydrophobicity of the molybdenite surface, creating a substantial wettability difference that facilitates effective copper–molybdenum flotation separation. Statistical error analysis and significance testing of contact angle measurements were conducted, with detailed results presented in Table 7 and Table 8. The analysis revealed consistently low standard deviations across all datasets (s < 1.04), confirming the high reproducibility of experimental procedures. Notably, statistically significant differences in contact angles were observed between chalcopyrite and molybdenite samples subjected to different treatment methods (*p* < 0.001). These findings demonstrate that MMO induces distinct levels of surface wettability modification in the two minerals, with chalcopyrite exhibiting a more pronounced response compared to molybdenite.

### 2.3. FT-IR Measurements

**Figure 5:**
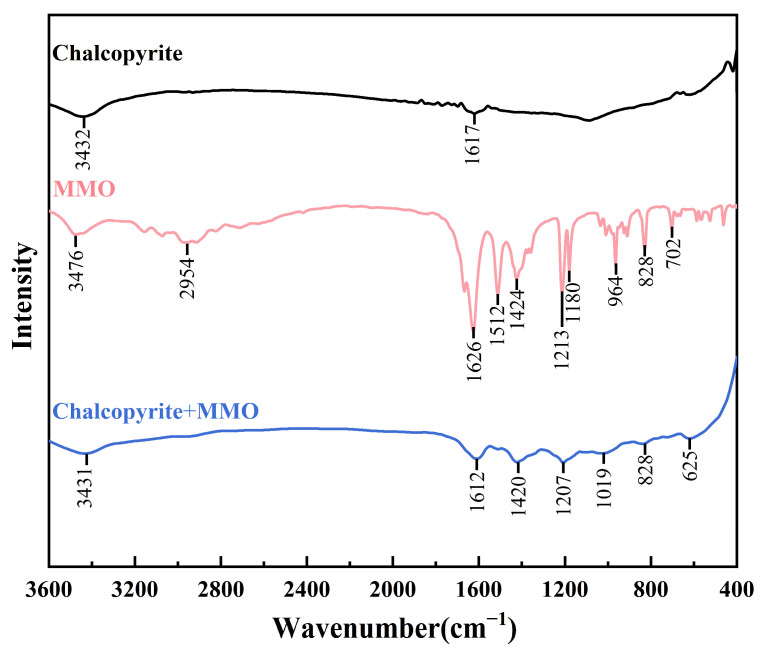
FT-IR spectra of MMO, treated and untreated chalcopyrite samples by MMO.

FT-IR analysis was conducted on chalcopyrite samples before and after treatment with MMO, and the results are presented in Figure 5. The untreated chalcopyrite sample exhibited two broad peaks near 3432 cm^−1^ and 1617 cm^−1^, attributed to the absorption of water molecules during the KBr method test. In the FTIR spectrum of MMO, the characteristic peaks at 3476 cm^−1^ and 2954 cm^−1^ correspond to N-H stretching vibrations and C-H stretching vibrations, respectively. Additionally, the peaks at 1626 cm^−1^, 1512 cm^−1^, 1424 cm^−1^, 1213 cm^−1^, and 1180 cm^−1^ are associated with C=C stretching vibrations, N-H in-plane bending vibrations, C-N stretching vibrations or C-H in-plane bending vibrations, C-O stretching vibrations, and C-S stretching vibrations, respectively. The peaks at 964 cm^−1^, 828 cm^−1^, and 702 cm^−1^ are related to C-H out-of-plane bending vibrations or N-H out-of-plane bending vibrations. For chalcopyrite samples treated with MMO, several characteristic peaks of MMO were detected compared to the untreated samples, indicating that MMO can adsorb onto the surface of chalcopyrite.

### 2.4. ToF-SIMS Measurement

**Figure 6:**
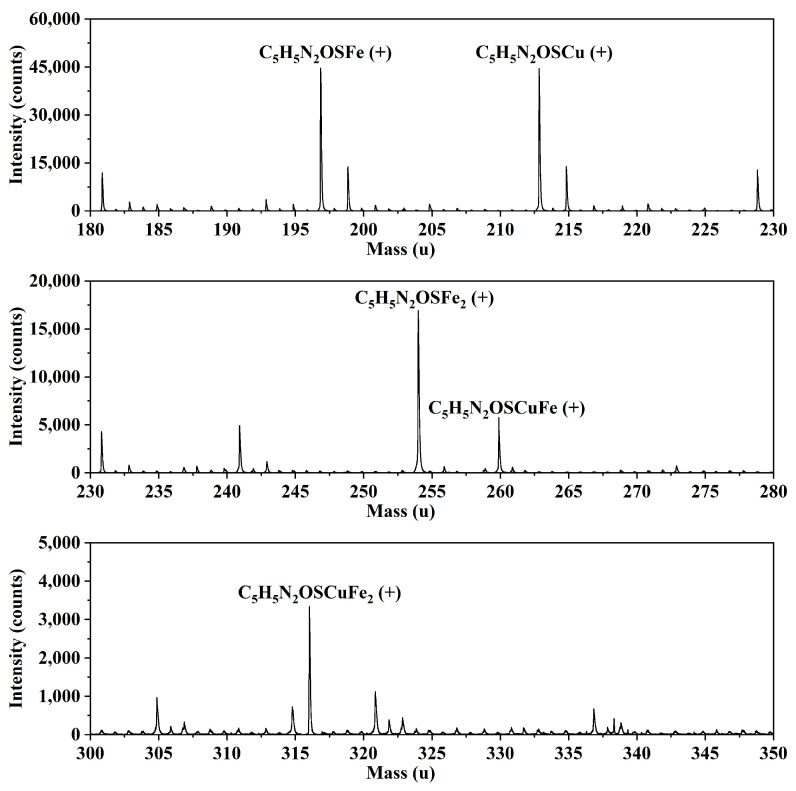
TOF-SIMS spectra (positive ions) of treated chalcopyrite samples by MMO.

Time-of-flight secondary ion mass spectrometry (TOF-SIMS) is a highly sensitive analytical technique widely utilized to study the adsorption of flotation reagents on mineral surfaces [28,29,30,31,32]. Figure 6 presents the positive ion mass spectrum of the chalcopyrite sample following MMO adsorption. The results reveal the formation of ions such as C_5_H_5_N_2_OSFe(+), C_5_H_5_N_2_OSCu(+), C_5_H_5_N_2_OSFe_2_(+), C_5_H_5_N_2_OSCuFe(+), and C_5_H_5_N_2_OSCuFe_2_(+) on the chalcopyrite surface. The presence of these ion fragments suggests that MMO tends to remove one hydrogen atom before adsorption on the chalcopyrite surface. This indicates that there may be 1–3 adsorption sites available on the chalcopyrite surface, potentially involving both Cu and Fe as binding sites.

### 2.5. Zeta Potential Measurement

**Figure 7:**
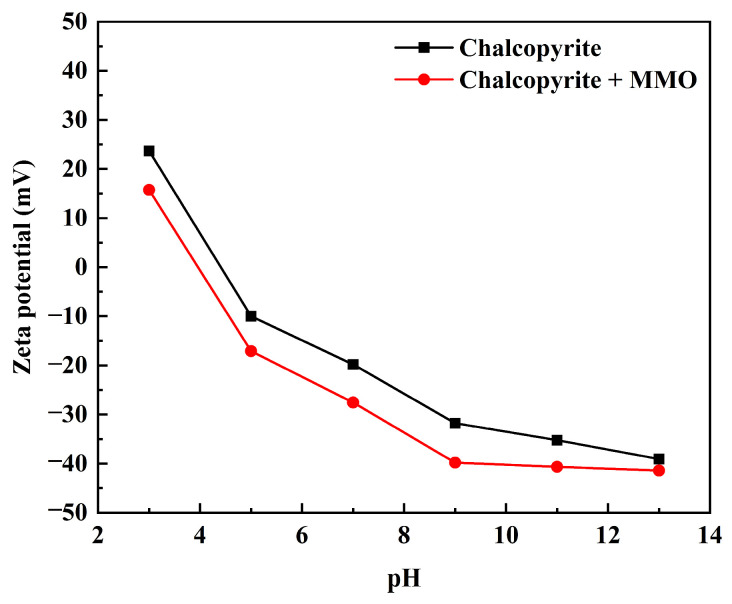
The zeta potential variations of untreated and MMO-treated chalcopyrite samples across varying pH conditions.

The zeta potential variations of untreated and MMO-treated chalcopyrite samples across varying pH conditions are presented in Figure 7. The application of MMO induced a substantial reduction in zeta potential values throughout the entire pH spectrum, indicating interaction between MMO molecules and negatively charged components on the mineral surface. A pronounced zeta potential decline observed at pH = 3, despite the highly acidic environment, confirms the preservation of anionic characteristics in MMO molecules, thereby ruling out significant protonation or chemical bond cleavage. Furthermore, the sustained negative charge enhancement at pH = 9 aligns with the inherent resistance of thiol (-SH) groups to alkaline hydrolysis. Comprehensive analysis reveals that performance discrepancies of MMO under different pH conditions originate from surface charge-mediated competitive adsorption mechanisms rather than structural degradation processes.

### 2.6. XPS Measurements

**Figure 8:**
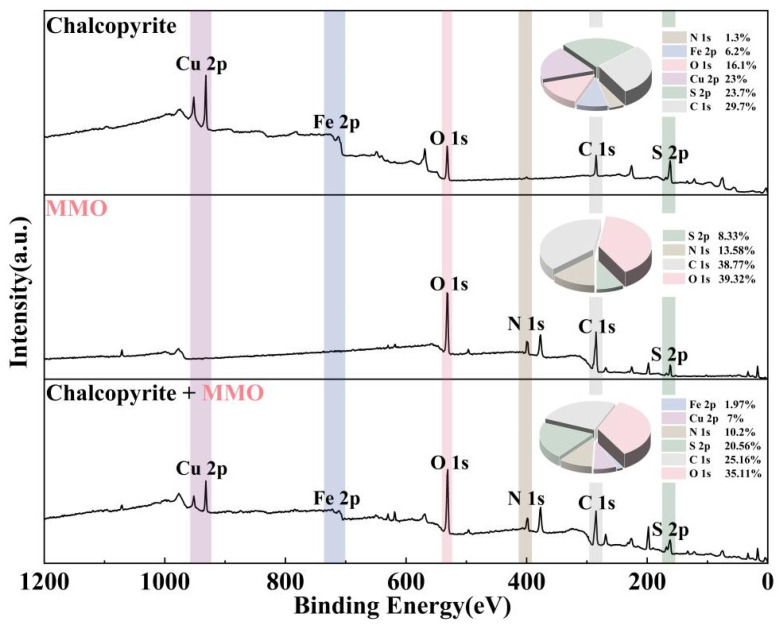
XPS spectra of MMO, treated and untreated chalcopyrite samples by MMO.

XPS analysis can provide further insight into the adsorption mechanism of the depressant MMO on the chalcopyrite surface [33,34,35]. Figure 8 displays the XPS spectra of MMO and chalcopyrite samples before and after MMO adsorption. After adsorption, a distinct N 1s orbital peak appeared on the chalcopyrite surface. Additionally, there was a significant increase in the proportion of O 1s orbital atoms, indicating that MMO was successfully adsorbed onto the chalcopyrite surface.

**Figure 9:**
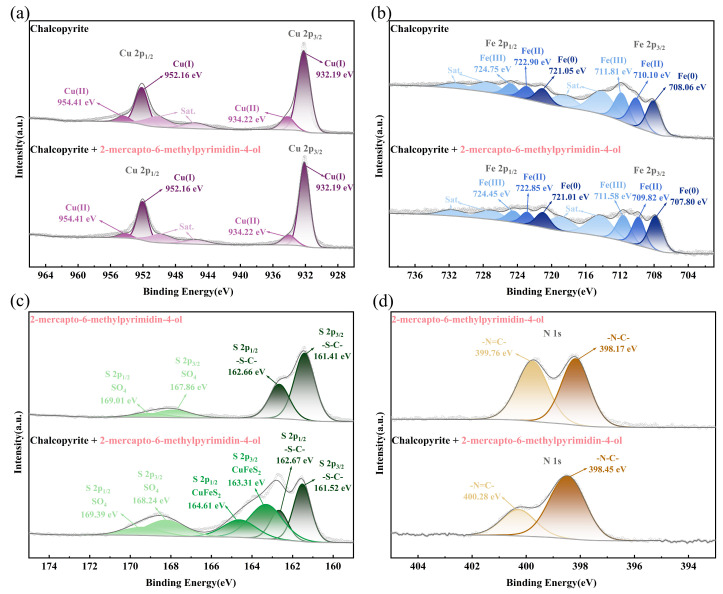
XPS spectra of MMO, treated and untreated chalcopyrite samples by MMO: (**a**) Cu 2p; (**b**) Fe 2p; (**c**) S 2p; and (**d**) N 1s.

Figure 9a,b illustrates the XPS spectra of the Cu 2p and Fe 2p peaks of the chalcopyrite sample before and after MMO adsorption. In the untreated chalcopyrite, the Cu 2p3/2 orbital displays a prominent Cu(I) peak at 932.19 eV, a weaker Cu(II) peak at 934.22 eV, and two very faint satellite peaks. The Fe 2p3/2 orbital is divided into a Fe(0) peak at 708.06 eV, a Fe(II) peak at 710.10 eV, and a Fe(III) peak at 711.81 eV, along with two strong satellite peaks that show little variation in intensity. After MMO adsorption, the binding energy of the Cu(I) peak in the Cu 2p3/2 orbital shifted to 932.12 eV, while the binding energy of the Cu(II) peak shifted to 934.04 eV, representing decreases of 0.07 eV and 0.18 eV, respectively. Similarly, the binding energy of the Fe(0) peak in the Fe 2p3/2 orbital shifted to 707.80 eV, the Fe(II) peak to 709.82 eV, and the Fe(III) peak to 711.58 eV, corresponding to decreases of 0.26 eV, 0.28 eV, and 0.23 eV, respectively. These decreases in binding energy after adsorption indicate an increase in electron cloud density, suggesting that the Cu and Fe orbitals have gained electrons provided by MMO.

Figure 9c,d presents the XPS spectra of the S 2p and N 1s peaks of the chalcopyrite sample after MMO adsorption. In MMO, the S 2p3/2 orbital displays a high-intensity peak at 161.41 eV and a weaker peak at 167.86 eV, attributed to the S-C group and the SO_4_ group resulting from slight oxidation, respectively. The N 1s orbital is characterized by two peaks with minimal intensity variation: the peak at 398.17 eV corresponds to the N-C group, while the peak at 399.76 eV is associated with the N=C group. After MMO adsorption, the binding energy of the S-C group in the S 2p3/2 orbital shifted to 161.52 eV, and the binding energy of the SO_4_ group shifted to 168.24 eV, increasing by 0.11 eV and 0.38 eV, respectively. Additionally, a new peak attributed to sulfur in chalcopyrite was detected at 163.31 eV. The binding energy of the N-C group in the N 1s orbital shifted to 398.45 eV, and the binding energy of the N=C group shifted to 400.28 eV, representing increases of 0.28 eV and 0.52 eV, respectively. These increases in binding energy after adsorption indicate a decrease in electron cloud density, suggesting that the S and N orbitals of MMO donate electrons to the chalcopyrite surface.

Analysis of the XPS results indicates that the adsorption of MMO on the chalcopyrite surface primarily occurs through the S and N atoms of MMO donating electrons to the copper and iron on the chalcopyrite surface. This finding is consistent with the results obtained from the TOF-SIMS analysis.

### 2.7. DFT Simulations

#### 2.7.1. Molecular Structure of Reagents

**Figure 10:**
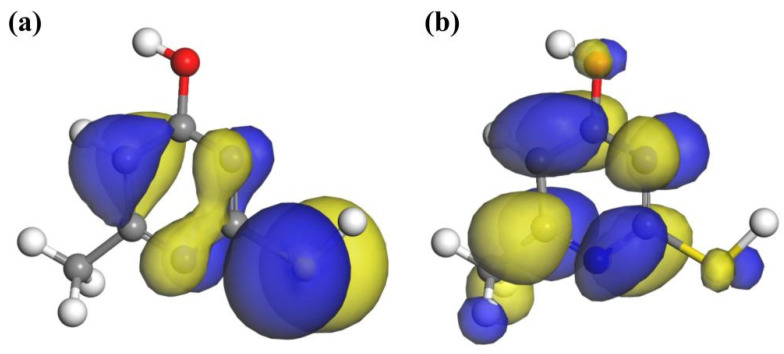
The frontier molecular orbitals of MMO: (**a**) HOMO; (**b**) LUMO.

The frontier orbital theory posits that the properties of a molecule are primarily determined by its highest occupied molecular orbital (HOMO) and lowest unoccupied molecular orbital (LUMO). Analyzing the frontier orbital energy of a collector can help predict its interaction with mineral surfaces [36,37]. The frontier orbital distribution of the MMO molecule is illustrated in Figure 10. The HOMO orbital reflects the molecule’s ability to donate electrons. The positively charged Cu^+^ and Fe^3+^ ions on the chalcopyrite surface are inclined to interact with atoms exhibiting significant HOMO contributions. As shown in the figure, the HOMO orbital of MMO is predominantly distributed over the N and S atoms, taking the form of a π orbital. Consequently, both N and S atoms may adsorb onto the chalcopyrite surface.

In chalcopyrite, the Cu^+^ and Fe^3+^ ions are situated in a tetrahedral field, causing their d orbital energy levels to split into π orbitals, with the d orbital electrons behaving as π electrons. If the interacting atoms within the collector molecule possess empty π orbitals, the Cu^+^ and Fe^3+^ ions can donate their d orbital electrons to these empty π orbitals, forming π backbonding. Conversely, the LUMO orbital indicates the molecule’s capacity to accept electrons. The O, N, and S atoms in the MMO molecule each have LUMO orbitals shaped as π orbitals, making them likely candidates to accept π electrons from the π orbitals of Cu^+^ and Fe^3+^ ions, thereby facilitating the formation of π backbonding with the chalcopyrite surface.

The calculated properties of each active atom in the MMO molecule are presented in Table 9. The Cu^+^ and Fe^3+^ ions on the chalcopyrite surface are positively charged and tend to interact with atoms that can donate more electrons or exhibit greater electronegativity. In the MMO molecule, the N1, N2, S, and O atoms are all negatively charged. The Fukui function measures the sensitivity of charge density based on electron gain and loss, allowing for the assessment of nucleophilicity and electrophilicity of the molecule or atom. The nucleophilicity index characterizes the ability to provide electrons, with the nucleophilicity order of the N1, N2, S, and O atoms in the MMO molecule being S > N2 > N1 > O.

These data indicate that the S atom is the most readily adsorbed onto the chalcopyrite surface, followed by the N atom. Additionally, analysis of atomic polarizability reveals that the polarizability of the S atom is significantly greater than that of the N and O atoms, resulting in stronger dispersion and induction forces that facilitate interaction with the chalcopyrite surface. Conversely, the O atom exhibits the lowest atomic polarizability, leading to weaker dispersion and induction forces, which may result in a relatively weak interaction with the chalcopyrite surface.

#### 2.7.2. Adsorption on the Surface of Chalcopyrite

**Figure 11:**
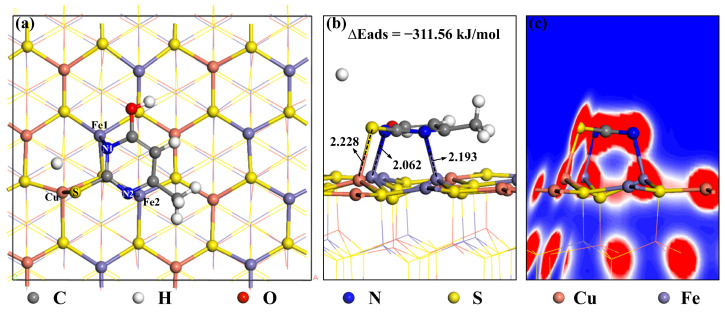
Adsorption models of MMO on a chalcopyrite surface: (**a**) top view; (**b**) main view; and (**c**) electron density diagram.

Figure 11a,b presents the calculated adsorption model of MMO on the chalcopyrite surface. During the adsorption process, the S atom of the MMO molecule loses one H ion and binds to the Cu ion on the chalcopyrite surface, while the N1 and N2 atoms adsorb onto the Fe1 and Fe2 ions, respectively. After adsorption, the bond lengths are measured as follows: N1-Fe1 is 2.062 Å, N2-Fe2 is 2.193 Å, and S-Cu is 2.228 Å—all of which are smaller than their atomic radii, indicating that adsorption can occur. The adsorption energy of this process is notably high at −311.56 kJ/mol. This is attributed to the three atoms in the MMO molecule being adsorbed onto three different sites on the chalcopyrite surface, forming a stable chelate structure with strong adsorption. Figure 11c displays the electron density diagram of MMO after adsorption on the chalcopyrite surface. The N1, N2, and S atoms of MMO show substantial electron cloud overlap with the Fe1, Fe2, and Cu ions on the chalcopyrite surface, indicating a strong covalent interaction. This adsorption configuration is further supported by the TOF-SIMS characterization results mentioned earlier.

The Mulliken population value of a bond reflects the strength of its ionic and covalent characteristics. A larger population value indicates a more covalent bond, while a smaller value suggests a more ionic bond. The Mulliken population calculations for the bonds in the MMO molecule adsorbed on the chalcopyrite surface are presented in Table 10. Analysis of the data reveals that although the S-Cu bond has the longest bond length, it exhibits the largest population value, indicating the strongest covalency. This is likely due to the greater polarizability of the S atom. Additionally, the N1-Fe1 and N2-Fe2 bonds, along with the S-Cu bond, demonstrate strong covalency, stable bonding, and robust adsorption characteristics.

**Figure 12:**
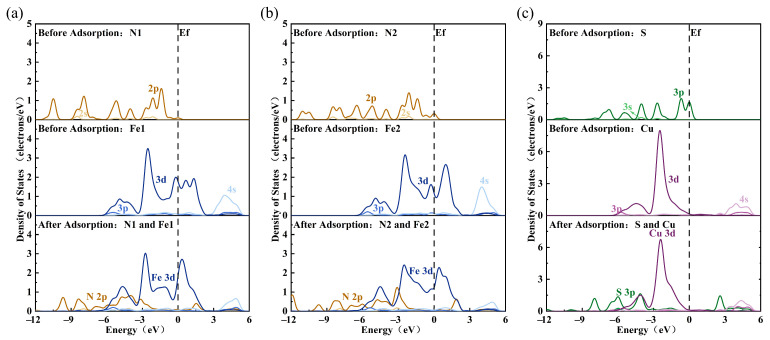
The PDOS of MMO adsorbed on chalcopyrite surface: (**a**) Ni-Fe1; (**b**) N2-Fe2; and (**c**) S-Cu.

Figure 12 illustrates the partial density of states (DOS) of N1, N2, and S atoms, along with the Fe1, Fe2, and Cu ions they adsorb after MMO is attached to the chalcopyrite surface. The figure shows that during the adsorption process, the 2p orbital of the N atoms interacts with the 3d orbital of the Fe ions, while the 3p orbital of the S atoms interacts with the 3d orbital of the Cu ions. After the N1 and N2 atoms adsorb onto the Fe1 and Fe2 ions, the 2p orbital of the N atoms shifts significantly to a deeper energy level, overlapping with the DOS of the Fe 3d orbital within the range of −7 eV to −2 eV. The DOS peak value of the Fe 3d orbital decreases, the distribution range expands, and electron delocalization increases, leading to a relatively stable interaction. Similarly, after the S atoms adsorb onto the Cu ions, the 3p orbital of the S atoms shifts to a deeper energy level, overlapping with the DOS of the Cu 3d orbital in the range of −7 eV to −1 eV. Here, the DOS peak value of the Cu 3d orbital decreases, electron delocalization increases, and a hybrid peak appears near −4 eV for the two orbitals, indicating a stronger adsorption effect. Post-adsorption, neither the S 3p orbital nor the Cu 3d orbital crosses the Fermi level, remaining in a relatively stable state, while the Fe 3d orbital still crosses the Fermi level, indicating that its electronic state remains active. Therefore, the adsorption of S atoms on Cu ions is more stable than that of N atoms on Fe ions.

**Figure 13:**
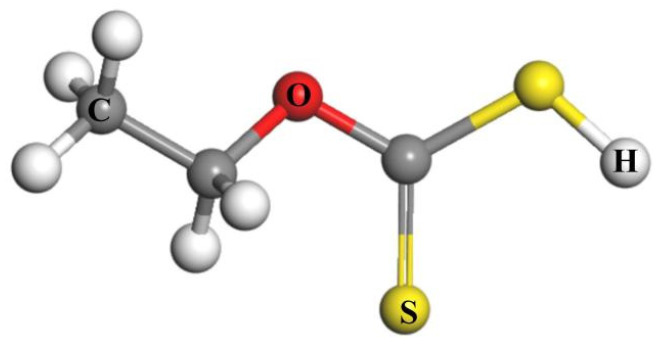
Molecular structure model of ethyl xanthate.

**Figure 14:**
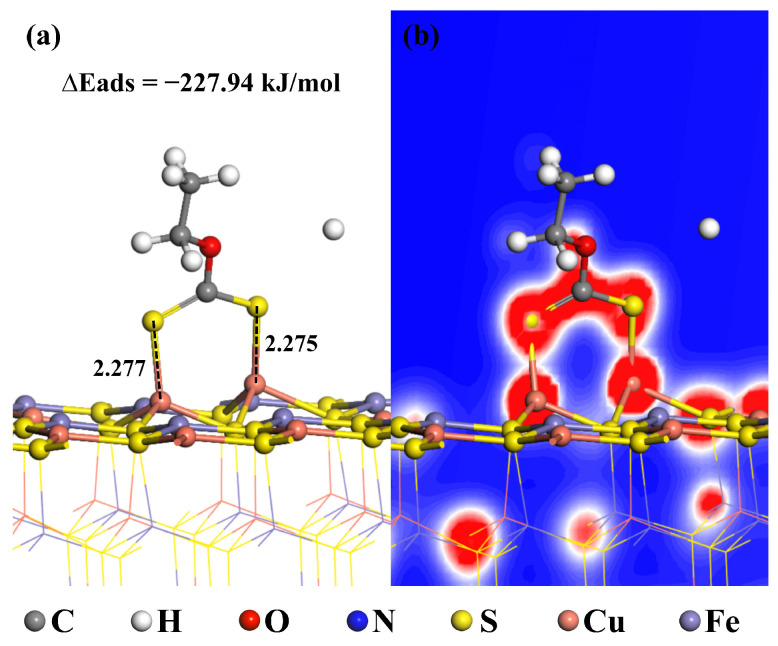
Adsorption models of ethyl xanthate on chalcopyrite surface: (**a**) main view; (**b**) electron density diagram.

To further investigate the mechanism by which the depressant MMO affects the flotation recovery of chalcopyrite, simulation calculations were performed on the adsorption of the collector ethyl xanthate on the chalcopyrite surface. The molecular model of ethyl xanthate is depicted in Figure 13, while the adsorption model is shown in Figure 14a. During the adsorption process, the two S atoms of the ethyl xanthate molecule adsorb onto different Cu ions on the chalcopyrite surface, with bond lengths after adsorption measuring 2.277 Å and 2.275 Å, both of which are smaller than their atomic radii, indicating successful adsorption. The adsorption energy for this process is −227.94 kJ/mol, which is lower than the −311.56 kJ/mol energy associated with MMO adsorption.

Figure 14b presents the electron density diagram following the adsorption of ethyl xanthate on the chalcopyrite surface. It is evident that the S atoms of ethyl xanthate exhibit a significant overlap of electron clouds with the Cu ions; however, this overlap is less pronounced compared to the electron cloud interactions observed between the N atoms and S atoms with the Fe and Cu ions during MMO adsorption, suggesting that the covalency is relatively weak. While ethyl xanthate can be stably adsorbed on the chalcopyrite surface, it is unlikely to interact through competitive adsorption after MMO has adhered. Consequently, MMO effectively depresses the flotation of chalcopyrite in the presence of a collector.

**Figure 15:**
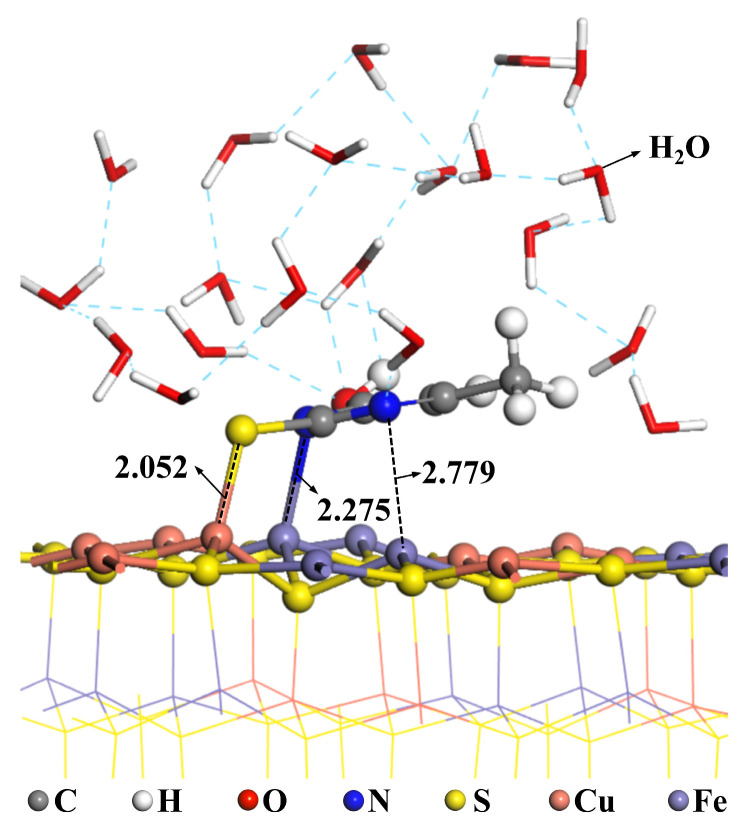
Dynamic adsorption models of MMO on chalcopyrite surface.

Flotation occurs in water, making it essential to consider the impact of water molecules on the adsorption of reagents. A dynamics method was employed to simulate the structure of MMO adsorbed on the chalcopyrite surface. The dynamics simulation was conducted at a temperature of 25 °C, with a calculation duration of 50 ps and a system comprising 20 water molecules. The results are displayed in Figure 15. In the presence of water molecules, the N1-Fe1 bond length increased from 2.062 Å to 2.275 Å, and the N2-Fe2 bond length rose from 2.193 Å to 2.779 Å. Conversely, the S-Cu bond length decreased from 2.228 Å to 2.052 Å. Notably, the S-Cu bond length is significantly reduced, while the N1-Fe1 bond length is considerably increased, leading to the breaking of the N2-Fe2 bond. This indicates a transition of MMO from triple bond adsorption to double bond adsorption, further confirming that S-Cu bond adsorption is more stable than N-Fe bond adsorption. After MMO adsorption, the 20 water molecules in the system become distributed across the chalcopyrite surface, forming hydrogen bonds with one another. Consequently, the chalcopyrite surface exhibits strong hydrophilicity, allowing MMO to effectively depress the flotation of chalcopyrite.

The adsorption mechanism of MMO on chalcopyrite surfaces involves two critical interactions: (1) covalent bonding between sulfur atoms in the thiol groups of MMO and copper ions on the chalcopyrite surface, as well as covalent bonding between nitrogen atoms in the pyrimidine ring and iron ions on the mineral surface, which is validated by TOF-SIMS analysis and XPS spectra; (2) hydrogen bonding between hydroxyl groups of adsorbed MMO and free water molecules, resulting in enhanced surface hydrophilicity. This dual interaction model explains both the strong depression effect of MMO on chalcopyrite and its high selectivity: molybdenite lacks reactive copper sites necessary for analogous bonding, thereby remaining unaffected during flotation.

### 2.8. Actual Ore Flotation Experiments

To evaluate the performance of MMO as a chalcopyrite depressant in Cu-Mo flotation separation, comparative experiments were conducted with traditional depressants Na_2_S and NaCN under controlled conditions (ambient temperature 25 °C, pH ≈ 7) using actual ore samples. As shown in Table 11, when MMO was employed, the molybdenum concentrate grade significantly increased to over 20% (approximately 1.5% higher than the Na_2_S and NaCN groups), while the copper grade decreased notably. The copper recovery in tailings was also markedly higher compared to the control groups, indicating superior selectivity of MMO for Cu-Mo separation. Furthermore, in contrast to Na_2_S (requiring high dosages) and NaCN (high toxicity), MMO demonstrated potential as an environmentally friendly alternative depressant.

## 3. Materials and Methods

### 3.1. Materials and Reagents

The pure chalcopyrite and molybdenite samples used in the microflotation experiments were collected from a chalcopyrite mine in Yunnan, China. Following hand selection, crushing, ball milling, and screening, samples with a particle size fraction of −74 µm +38 µm were obtained. The chemical composition of the samples was analyzed using chemical multiphase analysis. The grade of chalcopyrite was found to be higher than 96%, while the grade of molybdenite exceeded 98%, both of which met the requirements for the microflotation experiments.

The depressant MMO was purchased from Bide Pharmatech Ltd., Shanghai, China. The collector, ethyl xanthate; the frother, methyl isobutyl carbinol (MIBC); and the pH modifiers, sodium hydroxide and hydrochloric acid, were all sourced from Shanghai Aladdin Biochemical Technology Co., Ltd., Shanghai, China. Additionally, all water used in the experiments was deionized water.

For industrial-scale flotation validation, copper–molybdenum bulk concentrate samples were acquired from operational copper concentrates in the same mining region. Particle size distribution and multi-element composition are respectively detailed in Table 12 and Table 13.

### 3.2. Microflotation Experiments

The XFGCII-5 laboratory aerated hanging tank flotation machine (Jilin Province Jitan machinery Co., Ltd., Jilin, China) was employed to conduct micro-flotation experiments on pure chalcopyrite and molybdenite, with a fixed impeller speed of 1700 r·min^−1^. A total of 2 g of ore sample was placed in a flotation cell containing 50 mL of distilled water. The pH modifier, collector, depressant, and frother were added sequentially, with stirring times of 3 min for each of the first three additions, followed by 2 min for the frother. After aeration, the foam product was collected every 10 s over a total collection period of 3 min. The concentrate and tailings were then filtered, dried, and weighed to calculate the recovery rate.

### 3.3. Contact Angle Measurements

The contact angle of water droplets on the surfaces of chalcopyrite and molybdenite, both before and after treatment with depressants and collectors, was measured using an optical video-based DSA100E contact angle measuring instrument (KRÜSS Scientific Instruments (Shanghai) Co., Ltd., Shanghai, China). Several samples of chalcopyrite and molybdenite, approximately 2.0 cm × 2.0 cm × 0.5 cm in size, were prepared, polished with sandpaper of varying grits, ultrasonically cleaned in deionized water, and finally dried in a vacuum oven. Some of the dried samples were immersed in a 10^−3^ mol/L depressant solution for 10 min and gently rinsed with deionized water. Another set of chalcopyrite and molybdenite samples was first immersed in a 10^−3^ mol/L collector solution for 10 min, gently rinsed with deionized water, and then immersed in a 10^−3^ mol/L depressant solution for an additional 10 min, followed by another gentle rinse with deionized water. All samples were dried again in a vacuum oven before testing. Each measurement was repeated at least three times to minimize measurement errors.

### 3.4. FT-IR Measurements

The FT-IR spectra of MMO, as well as MMO-treated and untreated chalcopyrite samples, were characterized in the range of 400–4000 cm^−1^ using the KBr method with a Fourier transform spectrometer (Nicolet iS50, Thermo Fisher Scientific Inc., Shanghai, China). A 2 g sample of chalcopyrite, ground to −2 μm, was mixed with 50 mL of water, and a 10^−3^ mol/L depressant was added. After stirring for 20 min, the ore sample was filtered and washed three times. The mineral particles were then collected, vacuum dried, and finally mixed and ground with KBr before measuring the FT-IR spectrum.

### 3.5. TOF-SIMS Measurements

The surface chemical composition analysis of chalcopyrite before and after MMO treatment was performed using a time-of-flight secondary ion mass spectrometer (ION-TOF.SIMS 5, Beijing IONTOF Technology Co., Ltd., Beijing, China) equipped with a Bi^3+^ ion source. Sample preparation was consistent with that used in the FT-IR measurements. The primary ion beam voltage was set to 30 keV, with a current of 0.5 pA, and the mass range was configured between 0 and 500 amu.

### 3.6. Zeta Potential Measurements

Zeta potential measurements were conducted on chalcopyrite samples treated with and without MMO across varying pH conditions using a Zetasizer Nano ZS90 analyzer (Shanghai Spectris Instrumentation and Systems Ltd., Shanghai, China). For each experimental group, 2 g of chalcopyrite powder was dispersed in 100 mL of 10^−4^ mol/L NaCl solution, with or without the addition of 10^−3^ mol/L depressant solution. After stirring the mixture for 5 min, the pH was adjusted to target values using diluted HCl or NaOH solutions. The zeta potential of the stirred suspension was measured by sampling the supernatant, with a minimum of three independent replicates per pH condition to ensure statistical reliability.

### 3.7. XPS Measurements

To better understand the adsorption mechanism of MMO on the surface of chalcopyrite, X-ray photoelectron spectroscopy (XPS) was conducted on MMO, MMO-treated, and untreated chalcopyrite samples using a Thermo Fisher Scientific K-Alpha photoelectron spectrometer, Thermo Fisher Scientific Inc., Shanghai, China. Sample preparation was consistent with that used in the FT-IR test. XPS spectra were collected in the range of 0 eV to 1200 eV, with at least three scans performed for each sample. The calibration energy of the spectrometer was set to 100 eV, with a step size of 1.0 eV. The binding energy (B.E.) of 284.8 eV was assigned to C1s for spectrometer calibration.

### 3.8. DFT Simulations

**Figure 16:**
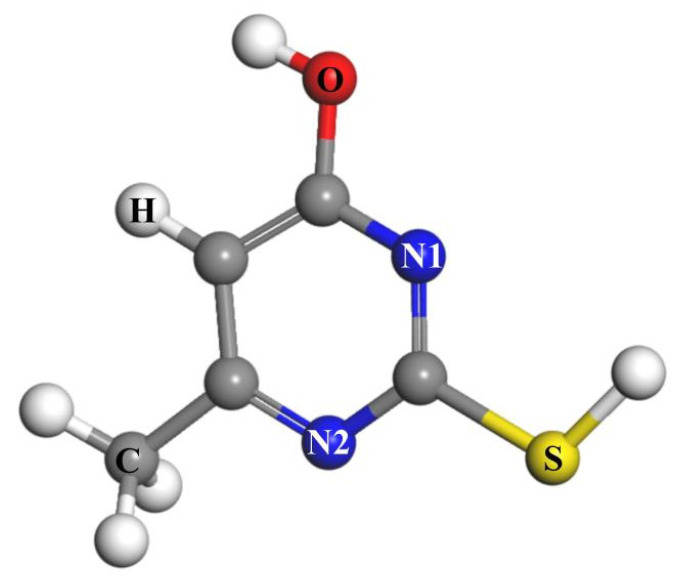
Molecular structure model of MMO.

This study implemented density functional theory (DFT) calculations using the Hartree-Fock (HF) functional in the DMol3 module and the GGA-PW91 functional in the CASTEP module of the Materials Studio 2017 software. The HF functional in the DMol3 program is known for its high precision and is particularly suitable for studying molecular structures and properties [38]. It was utilized to investigate the frontier orbitals, partial density of states, and charge distribution of MMO, as well as to identify the bonding atoms. The molecular model of the MMO drug was established, and the optimized drug structure model is illustrated in Figure 16.

The experimental lattice parameters of chalcopyrite were determined as a = b = 5.289 Å and c = 10.423 Å [39]. Table 14 summarizes the calculated lattice constants using the CASTEP module under different exchange-correlation functionals, along with their deviations from experimental values. Analysis revealed that the GGA-PW91 functional exhibited superior accuracy, with axial deviations of 2.84% (a/b-axis) and 2.76% (c-axis), establishing it as the optimal functional for subsequent calculations. Table 15 details the lattice parameters of chalcopyrite optimized with the GGA-PW91 functional at varying cutoff energies. The computational results demonstrated a gradual convergence toward experimental values as the cutoff energy increased, with the discrepancy between calculated and experimental parameters progressively diminishing. Beyond 400 eV, however, the rate of error reduction attenuated significantly. Balancing computational efficiency and accuracy, a cutoff energy of 400 eV was selected for final calculations.

**Figure 17:**
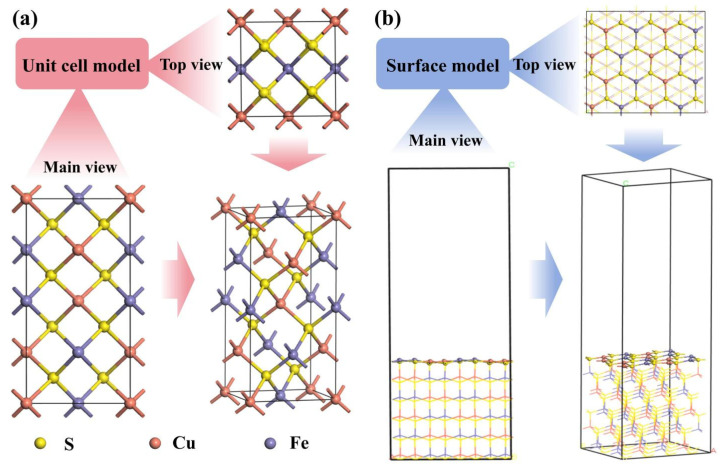
Calculation models of chalcopyrite: (**a**) unit cell model; (**b**) surface model.

**Figure 18:**
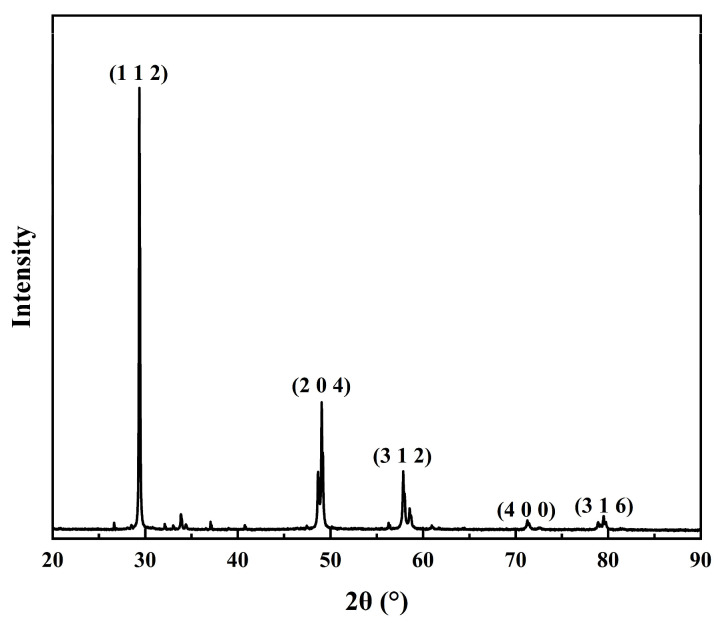
XRD characterization results of chalcopyrite.

The GGA-PW91 functional of the CASTEP program was used to study the adsorption of depressants on the chalcopyrite surface, analyzing the density of states of bonding atoms, charge density, and Mulliken population of bonds to assess bond strength. After optimizing the chalcopyrite unit cell using the GGA-PW91 functional in the CASTEP program, the resulting chalcopyrite unit cell model is shown in Figure 17a. The (112) surface is predicted to be the most stable surface based on the interatomic potential and DFT methods [40,41], which is further supported by our XRD characterization results (Figure 18); thus, it was selected for further study. The most stable unit cell surface model, derived from the optimized bulk structure, contains five atomic layers, with a vacuum layer thickness set to 20 Å. Following unit cell optimization, a (2 × 2 × 1) supercell geometry was modeled, resulting in the final surface model used for calculations, as illustrated in Figure 17b. During the calculation process, the cutoff energy was set to 300 eV to maintain a high level of accuracy for each calculation.

The adsorption energy is calculated as Equation (1):

(1) $${\Delta E}_{ads}=E_{adsorbates/surface}-E_{surface}-E_{adsorbates}$$

where E_ads_ denotes the adsorption energy, E_adsorbates/surface_ denotes the total energy of the reagent and the surface after adsorption, E_surface_ denotes the energy of the chalcopyrite surface before adsorption, and E_adsorbates_ denotes the energy of the reagent.

### 3.9. Actual Ore Flotation Experiments

In the actual ore flotation experiments, 100 g of ore sample was utilized per experimental run and introduced into a flotation cell containing 500 mL of deionized water. The flotation machine impeller maintained a constant rotational speed of 1700 rpm throughout the process. Reagents were administered following a sequential protocol: pH modifier, depressant, and frother, with respective agitation durations of 3 min for the pH modifier and depressant and 2 min for the frother. Following a 3 min flotation period, both concentrate and tailings products underwent filtration, desiccation, and subsequent grade analysis to calculate recovery rates.

## 4. Conclusions

1. The contact angle measurement results indicated that MMO substantially enhanced the hydrophilicity of the chalcopyrite surface while having a minimal effect on molybdenite. The increase in surface hydrophobicity of chalcopyrite treated with ethyl xanthate followed by MMO was not significant; in contrast, molybdenite experienced a considerable improvement in hydrophobicity after being treated with ethyl xanthate followed by MMO, leading to a notable difference in surface wettability. Consequently, MMO could serve as a depressant for chalcopyrite in copper–molybdenum separation flotation. Microflotation experiments corroborated this finding, showing that MMO effectively depressed chalcopyrite across a wide pH range while exerting little influence on the flotation recovery rate of molybdenite.
2. FT-IR, TOF-SIMS, and XPS tests and DFT simulation results revealed that MMO could adsorb onto the chalcopyrite surface. During the adsorption process, the N1, N2, and S atoms in the MMO molecule bonded with the Fe1, Fe2, and Cu ions on the chalcopyrite surface, respectively, resulting in the MMO molecule losing electrons while the chalcopyrite surface gained them. This configuration represented triple bond adsorption, forming a stable chelate structure with strong adsorption.
3. Dynamic simulations were employed to examine the effect of water molecules on the adsorption of MMO on the chalcopyrite surface. It was found that MMO remains stably adsorbed on the chalcopyrite surface in the presence of water. Additionally, water molecules were distributed close to the chalcopyrite surface and were interconnected by hydrogen bonds, indicating that MMO adsorption enhanced the hydrophilicity of the chalcopyrite surface, thereby depressing its flotation.

## Figures and Tables

**Table 1:** Error calculation results of flotation experiments investigating the effects on the flotation behaviors of chalcopyrite and molybdenite under various depressant dosages without collector.

Mineral	Dosage (×10^−5^ mol/L)	Recovery1 (%)	Recovery2 (%)	Recovery3 (%)	Recovery Average (%)	Standard Deviation	Error Bar
Chalcopyrite	0.5	57.26	60.31	59.78	59.12	1.63	59.12 ± 1.63
	1	16.23	16.98	17.89	17.03	0.83	17.03 ± 0.83
	1.5	9.98	9.07	9.93	9.66	0.48	9.66 ± 0.48
	2	4.03	4.51	4.16	4.23	0.24	4.23 ± 0.24
	2.5	0.59	0.47	0.44	0.50	0.08	0.5 ± 0.08
Molybdenite	0.5	69.45	64.23	66.77	66.82	2.63	66.82 ± 2.63
	1	61.33	61.90	63.76	62.33	1.22	62.33 ± 1.22
	1.5	57.84	58.35	60.10	58.76	1.14	58.76 ± 1.14
	2	57.49	55.86	53.55	55.63	1.97	55.63 ± 1.97
	2.5	52.43	54.26	55.95	54.21	1.76	54.21 ± 1.76

**Table 2:** Significance verification results of the effects on the flotation behaviors of chalcopyrite and molybdenite under various depressant dosages without collector.

Dosage (×10^−5^ mol/L)	*t*-Value	Degrees of Freedom (df)	*p*-Value Range	Significance (α = 0.05)
0.5	−4.33	3.4	*p* < 0.05	Significant
1	−29.02	3.28	*p* < 0.001	Significant
1.5	−29.8	3.78	*p* < 0.001	Significant
2	−31.72	3.35	*p* < 0.001	Significant
2.5	−33.94	3.63	*p* < 0.001	Significant

**Table 3:** Error calculation results of flotation experiments investigating the effects on the flotation behaviors of chalcopyrite and molybdenite under various depressant dosages.

Mineral	Dosage (×10^−5^ mol/L)	Recovery1 (%)	Recovery2 (%)	Recovery3 (%)	Recovery Average (%)	Standard Deviation	Error Bar
Chalcopyrite	3.5	66.86	68.33	67.32	67.5	0.75	67.5 ± 0.75
	6.5	60.3	61.36	61.69	61.12	0.7	61.12 ± 0.7
	20	44.15	48.94	46.95	46.68	2.4	46.68 ± 2.4
	50	23.01	21.17	22	22.06	0.92	22.06 ± 0.92
	60	11.98	10.45	11.51	11.31	0.77	11.31 ± 0.77
	80	10.63	9.96	9.02	9.87	0.83	9.87 ± 0.83
Molybdenite	3.5	89.78	86.94	88.52	88.41	1.43	88.41 ± 1.43
	6.5	84.96	85.63	85.20	85.26	0.34	85.26 ± 0.34
	20	80.11	81.57	81.21	80.96	0.77	80.96 ± 0.77
	50	72.46	74.65	72.64	73.25	1.12	73.25 ± 1.12
	60	70.01	70.96	71.54	70.84	0.77	70.84 ± 0.77
	80	66.96	69.78	68.25	68.33	1.41	68.33 ± 1.41

**Table 4:** Significance verification results of the effects on the flotation behaviors of chalcopyrite and molybdenite under various depressant dosages.

Dosage (×10^−5^ mol/L)	*t*-Value	Degrees of Freedom (df)	*p*-Value Range	Significance (α = 0.05)
3.5	−22.48	2.71	*p* < 0.001	Significant
6.5	−21.53	2.2	*p* < 0.001	Significant
20	−25.61	2.4	*p* < 0.001	Significant
50	−35.17	2.96	*p* < 0.001	Significant
60	−67.32	2.98	*p* < 0.001	Significant
80	−42.19	2.53	*p* < 0.001	Significant

**Table 5:** Error calculation results of flotation experiments investigating the effects on the flotation behaviors of chalcopyrite and molybdenite under various pH levels.

Mineral	pH	Recovery1 (%)	Recovery2 (%)	Recovery3 (%)	Recovery Average (%)	Standard Deviation (%)	Error Bar (%)
Chalcopyrite	3	95.34	96.28	97.01	96.21	0.84	96.21 ± 0.84
	5	13.88	14.56	14.06	14.17	0.34	14.17 ± 0.34
	7	11.88	11.03	11.02	11.31	0.47	11.31 ± 0.47
	9	1.13	1.21	1.22	1.19	0.05	1.19 ± 0.05
	11	0.66	0.78	0.69	0.71	0.06	0.71 ± 0.06
	13	0.51	0.52	0.44	0.49	0.04	0.49 ± 0.04
Molybdenite	3	90.06	91.58	90.61	90.75	0.78	90.75 ± 0.78
	5	73.49	72.75	71.69	72.64	0.90	72.64 ± 0.90
	7	69.93	70.96	71.63	70.84	0.86	70.84 ± 0.86
	9	68.88	69.06	67.54	68.49	0.76	68.49 ± 0.76
	11	65.46	64.91	65.03	65.13	0.28	65.13 ± 0.28
	13	61.98	62.97	60.99	61.98	1.03	61.98 ± 1.03

**Table 6:** Significance verification results of the effects on the flotation behaviors of chalcopyrite and molybdenite under various pH levels.

pH	*t*-Value	Degrees of Freedom (df)	*p*-Value Range	Significance (α = 0.05)
3	8.27	3.8	*p* < 0.01	Significant
5	−65.34	3.98	*p* < 0.001	Significant
7	−66.01	3.97	*p* < 0.001	Significant
9	−101.5	2.03	*p* < 0.001	Significant
11	−202.8	2.1	*p* < 0.001	Significant
13	−58.45	3.57	*p* < 0.001	Significant

**Table 7:** Error calculation results of contact angle measurements.

Mineral	Sample	Contact Angle1 (°)	Contact Angle2 (°)	Contact Angle3 (°)	Recovery Average (°)	Standard Deviation (°)	Error Bar (°)
Chalcopyrite	Untreated	56.7	58.6	58.4	57.9	1.04	57.9 ± 1.04
	Treated by MMO	7.8	7	6.8	7.2	0.5	7.2 ± 0.5
	Treated by ethyl xanthate and MMO	22.9	24.1	23.2	23.4	0.62	23.4 ± 0.62
Molybdenite	Untreated	66.8	67.3	66.9	67	0.25	67 ± 0.25
	Treated by MMO	61.3	61.5	61.9	61.6	0.31	61.6 ± 0.31
	Treated by ethyl xanthate and MMO	97.6	97.2	98.5	97.8	0.7	97.8 ± 0.7

**Table 8:** Significance verification results of contact angle measurements.

Sample	*t*-Value	Degrees of Freedom (df)	*p*-Value Range	Significance (α = 0.05)
Untreated	−14.68	2.33	*p* < 0.001	Significant
Treated by MMO	−87.6	2.03	*p* < 0.001	Significant
Treated by ethyl xanthate and MMO	−60.47	2.93	*p* < 0.001	Significant

**Table 9:** Calculation results of atomic properties in MMO.

Atomic	N1	N2	S	O
Mulliken charges	−0.594	−0.558	−0.42	−0.793
Fukui	0.054	0.091	0.352	0.045
Polarizabilities (a.u.)	7.343	7.165	18.913	4.948

**Table 10:** Mulliken population of the bonds after adsorption.

Bond	N1-Fe1	N2-Fe2	S-Cu
Mulliken population	0.24	0.28	0.47
Distance/Å	2.062	2.193	2.228

**Table 11:** The results of actual ore flotation experiments with different depressants.

Depressant	Dosage (g/t)	Concentrate			Tailings		
		Recovery (Mo/%)	Grade (Mo/%)	Grade (Cu/%)	Recovery (Cu/%)	Grade (Cu/%)	Grade (Mo/%)
Na_2_S	1000	98.71	16.12	9.42	76.72	23.28	0.114
NaCN	25	98.74	13.04	12.51	63.03	17.91	0.14
MMO	100	97.27	20.72	8.03	82.95	19.54	0.11

**Table 12:** The results of particle size analysis.

Particle Size (mm)	0.074	−0.037	−0.018	−0.009	−0.005	−0.002	Total
Productivity (%)	8.73	37.42	46.59	4.16	1.18	1.92	100

**Table 13:** The results of multi-element analysis.

Composition	Cu	Mo	Fe	Zn	Pb	Mn	S
Content (%)	23.8	0.24	31.9	0.06	0.05	0.03	36.1
Composition	Na_2_O	MgO	Al_2_O_3_	SiO_2_	K_2_O	CaO	Others
Content (%)	0.15	0.3	0.28	2.8	0.23	0.37	3.69

**Table 14:** Chalcopyrite lattice parameters after geometric optimization under different functionals.

Functional	Lattice Parameters/Å		Δa	Δc
	a = b	c		
BLYP	5.0975	9.9868	−3.62%	−4.18%
PW91	5.1388	10.1350	−2.84%	−2.76%
PBE	5.1337	10.1339	−2.94%	−2.77%
PBESOL	5.0981	9.6995	−3.61%	−6.94%
RPBE	5.0870	10.0561	−3.82%	−3.52%
WC	5.1037	9.7223	−3.50%	−6.72%
Exp.	5.289	10.423	0.00%	0.00%

**Table 15:** Test for plane-wave cutoff energy.

Cutoff Energy (eV)	Lattice Parameters/Å		Δa	Δc
	a = b	c		
280	5.1033	10.0868	−3.51%	−3.23%
320	5.1388	10.135	−2.84%	−2.76%
360	5.1599	10.1487	−2.44%	−2.63%
400	5.1783	10.1921	−2.09%	−2.22%
440	5.1802	10.1955	−2.06%	−2.18%
480	5.1837	10.2007	−1.99%	−2.13%
Exp.	5.289	10.423	0.00%	0.00%

## Data Availability

The original contributions presented in this study are included in the article. Further inquiries can be directed to the corresponding author(s).
